# Case of Cyanosis

**Published:** 1856-06

**Authors:** J. T. Jameson

**Affiliations:** South Otselic, Chenango County, N. Y.


### ART. IV.—*Case of Cyanosis.* By J. T. Jameson, M. D., South Otselic, Chenango Co., N. Y.

Mr. Editor: Should the following case of open foramen ovale, &c., be of sufficient importance, in connection with the interesting one of cyanosis recorded in your July number, to merit publication, it is at your service. The report of the case is confessedly very imperfect in all its details, having occurred many years ago in the practice of my preceptor during the period of my pupilage. I was present at the inspection of the body, but the brief history of the case was derived from him, and recorded at the time, together with the post-mortem appearances:

Miss G., æt. 18, has never enjoyed good health, being subject to dyspnœa, especially on making any exertion. The hands, nails, and lips, are remarkable on account of their bluish color. Has experienced pain occasionally in the region of the stomach, and about a week ago was taken with vomiting, which was allayed by small doses of calcined magnesia. On Saturday she dined out, and shortly after dinner began to feel unwell, and soon experienced violent pain in the situation of the stomach, and abdomen generally, which continued during Sunday, on the evening of which day she died. During Sunday the abdomen became tympanitic and very painful on pressure. The nature of the case was not clearly understood.

*Post-mortem.* The peritoneum was inflamed in different parts, and the small intestines matted together. There was considerable fluid in the abdominal cavity, exhaling a strong odor of the medicines taken by the patient during life. On opening the stomach, an ulcerated aperture capable of admitting the finger, with a thickened and dark-colored margin, was observed at the lesser curvature, and near to the cardiac orifice. A similar looking ulcer was also found at the greater curvature, but here the stomach had become adherent to the pancreas, which in this situation was enlarged and harder than natural, the adhesion thus formed having been obviously the means of prolonging the patient's life. The lungs were of a remarkable purple lilac color, but otherwise appeared to be healthy. The heart was larger than normal. The left auricle was much smaller than natural, whilst the right was judged to be larger. On examining the fossa ovalis, an aperture was observed capable of admitting the thumb.

*Remarks.* 1. It is somewhat astonishing that this patient should have enjoyed so good a degree of health up to the day before her death: so good as to be at a dinner party, having at this very time two large ulcers in her stomach, one of them just ready to perforate the peritoneal tunic, and actually doing so shortly after dining.

2. Had these ulcers any connection with the abnormal condition of the circulation; or was it only an accidental conjunction?

3. How explain the remarkable difference in the size of the two auricles, and the preternatural enlargement of the right?

4. The diagnosis in such a case, I mean in relation to the perforation of the stomach, is manifestly attended with considerable difficulty, and can at best, I should suppose, amount but to strong probability. How easy to mistake the case for one of spasm of the stomach, or pain caused by overloading the same, as, if I remember correctly, was done in the first instance, by my esteemed and talented preceptor.

